# Interactions between C-steel and blended cement in concrete under radwaste repository conditions at 80 °C

**DOI:** 10.1038/s41598-023-42645-6

**Published:** 2023-09-16

**Authors:** Margit Fabian, Otto Czompoly, Istvan Tolnai, Laurent De Windt

**Affiliations:** 1 Centre for Energy Research, Konkoly Thege st 29-33, 1121 Budapest, Hungary; 2 Centre for Geosciences and Geoengineering, Mines Paris, PSL University, 77300 Fontainebleau, France

**Keywords:** Environmental chemistry, Metals and alloys, Imaging studies

## Abstract

Deep geological repository is widely considered as the preferred solution for the final disposal of high-level radioactive waste. Investigation representative of the Hungarian disposal concept was conducted using mock-up diffusion cells to study the chemical changes of S235JR carbon steel canister and CEM II/B concrete of the Public Limited Company for Radioactive Waste Management under anerobic and water saturated conditions at 80 °C. Micro-Raman, Scanning Electron Microscopy-Energy Dispersive X-ray Spectroscopy, fluid and potentiometric analysis were performed over a period of 12 months. The analysis was supported by thermodynamic and reactive transport modeling using the HYTEC code. The findings revealed that a uniform corrosion process occurred, leading to rapid passivation of the C-steel with magnetite as the primary corrosion product. Modeling demonstrated that the increase in temperature to 80 °C and the chemical evolution of the concrete did not significantly affect the corrosion passivation process. Although the formation of Fe-siliceous hydrogarnets is thermodynamically possible at 80 °C, it did not jeopardize magnetite passivation. The results show that the passivation of the containers occurred under the test conditions and this is a promising result for further investigations.

## Introduction

All states that engage in any kind of nuclear application must consider the management of radioactive waste and make sure it is handled in a safe manner regarding the level of radioactivity and complying with national/international regulations. There is a broad consensus that the preferred method of ensuring the long-term safety for high level radioactive waste (HLW) is isolation in a deep geological repository (DGR), which will provide passive multibarrier isolation of radioactive materials. Achieving this goal requires both natural geological barriers and an engineered barrier system (EBS) with complementary safety functions, creating a robust system to enhance confidence in the protection that will be provided^1,2^. The EBS itself comprises a variety of sub-systems, such as the waste form (radioactive material immobilized in a host material), a corrosion resistant and mechanically stable container, a buffer/sealing system, and plugs. The EBS must be designed so that it will work with the natural barriers to meet the regulatory limits^3,4^. The vitrified HLW form in a steel canister is specifically designed for long term durability in storage and disposal^5,6^. The requirements for lifetime and integrity of the steel canister depend on the DGR concept and the geologic formation^7^. The radioactive decay of the HLW will generate heat for several centuries to thousands of years depending on the HLW type, which will lead to maximum temperatures at the canister/buffer interface around 90 °C^8^. After the sealing of the HLW disposal cell, oxygen will be rapidly consumed, and anoxic conditions will prevail^9^.

The Hungarian radioactive waste management company is designing a DGR in the Boda Claystone Formation (BCF) in West-Mecsek (SW-Hungary). The interface between carbon steel and CEM II-based concrete is a key issue in the design of a disposal cell for vitrified HLW in argillaceous rock formations for the Hungarian national waste disposal program^10,11^. The design relies on low carbon-steel (C-steel) containers containing the HLW encased in a prefabricated cylindrical concrete buffer material. The concrete, which originated from the Public Limited Company for Radioactive Waste Management (PURAM), is considered as the buffer material in the final disposal program in Hungary^12^. This concept presents many similarities with the super-container concept in Belgium and the Netherlands with a C-steel overpack encased in a cylindrical concrete buffer consisting of CEM I and limestone aggregates^13^. The present investigation will also help to optimize the Hungarian repository concept with the aim of ensuring its long-term safety.

C-steel is never in equilibrium with water even under anoxic conditions and it will be subject to several types of corrosion mechanisms, such as uniform corrosion and localized corrosion (e.g. pitting corrosion). The corrosion of C-steel in concrete has been widely investigated^14,15^, particularly focusing on aerated conditions and rather low temperatures in the framework of the durability of steel reinforced concrete in civil engineering. To our knowledge, the corrosion under elevated temperature and anoxic conditions representative of nuclear waste geological disposal has been much less studied in the literature. Recently, Chomat et al.^16^ performed experiments with steel coupons embedded in blended (CEM V/A) cement pastes in water-saturated and anoxic conditions at 50 °C for 3 years. A Fe-enriched layer formed directly at the surface of the steel did not increase in thickness with aging time. The measured corrosion rates were typical of a passive corrosion mechanism. Smart and coauthors investigated the anaerobic corrosion of carbon steel wire in aqueous solutions representative of young CEM I porewater at 25 and 80 °C^17^. The corrosion rate was initially high but rapidly fell below 0.1 µm/year probably due to magnetite passivation. Pally et al.^18^ have recently tested the anaerobic corrosion of metallic iron plates immersed in synthetic CEM I water at 80 °C. They demonstrated that a thin film of magnetite formed on the plates during the first immersion days followed by the formation of a compact layer of Fe-siliceous hydrogarnet (with passivation properties).

This study focuses on C-steel corrosion in contact with CEM II-based concrete at 80 °C to gain further information on the chemical-physical alteration of the steel/concrete interface for conditions representative of geologic disposal. Laboratory scale experiments were performed at a temperature of 80 °C, under water-saturated and anoxic conditions during 12 months. A set of complementary characterization methods was applied to the liquid and solid phases with the support of geochemical modeling.

## Materials and experiments

### Concrete and steel compositions

The concrete originated from the National Radioactive Waste Repository in Bátaapáti (Hungary). This underground repository ensures the final disposal of short-lived low- and intermediate-level solid or solidified radioactive waste of nuclear power plant origin^19^. The concrete was made of CEM II/B (18 wt.%), siliceous sand/gravel (54 wt.%), limestone flour (15 wt.%) and tap water (11 wt.%), as detailed in Table 1.

**Table 1:** Composition of concrete.

Materials		Density [kg/dm^3^]	Mass [kg]	Volume [l]
Main components				
Aggregates	Sand	2.7	920	347
	Gravel	2.7	230	87
Cement	CEM II/B-S 42.5 N	3.1	385	126
Limestone flour	Limestone flour	2.7	315	117
Water	Tap water	1.0	231	231
Minor components				
Fiberglass		2.6	1	0.5
Admixtures^33^	Sika ViscoCrete	1.1	5	5
	Sika Control-40	1.0	10	10
	Sika LPS A-94	1.0	1	1
	SikaFume	2.2	26	11
Total			2124	935

The steel container was made of untreated S235JR carbon steel. The chemical composition of the C-steel corresponded to ≤ 0.17 C, ≤ 0.3 Si, ≤ 1.4 Mn, 0.035 P, 0.035 S, ≤ 0.55 Cu, ≤ 0.012 N in wt.%.

### Design of the cell experiments

**Figure 1:**
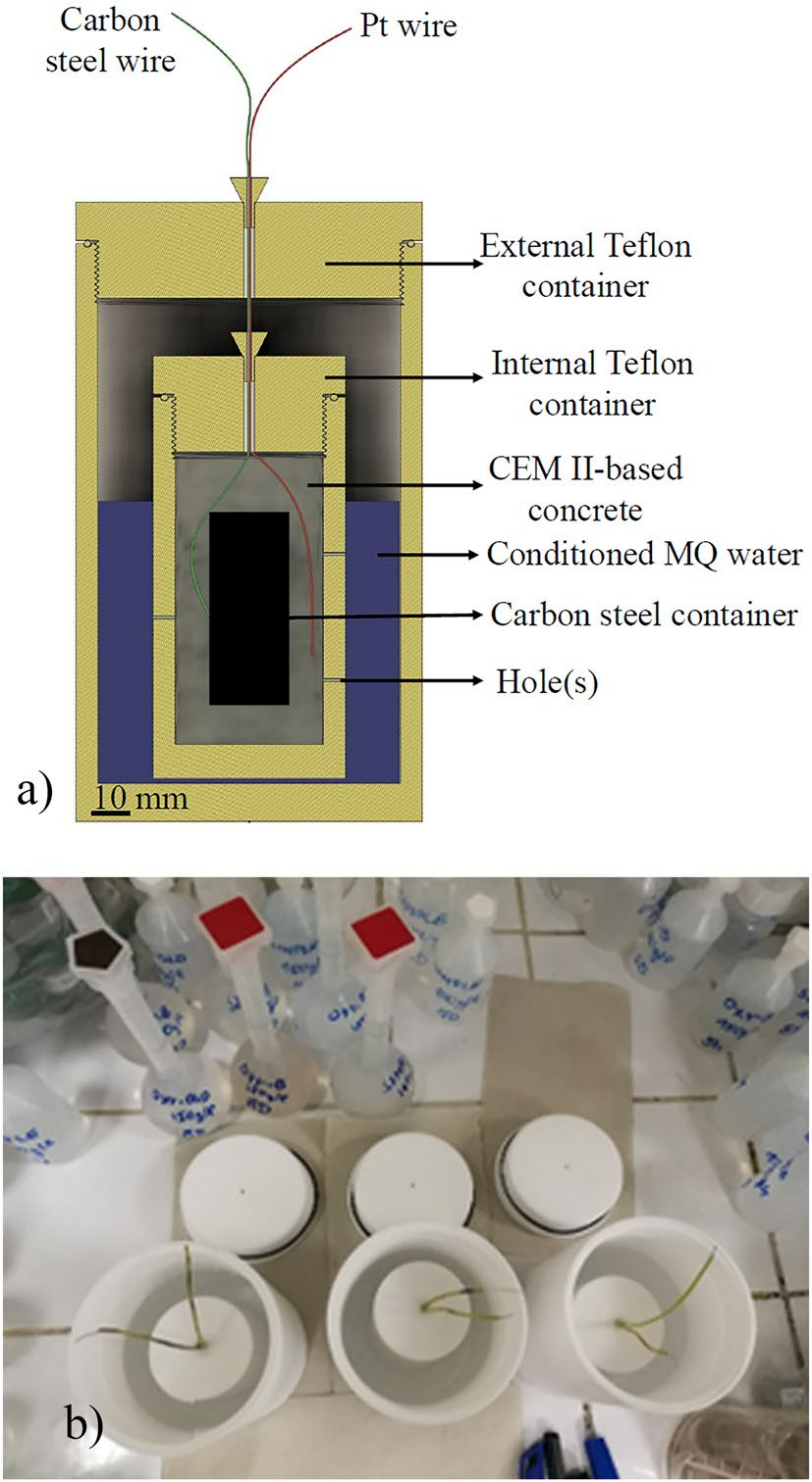
(**a**, **b**) Cross-section (**a**) and photograph (**b**) of the experimental setup in triplicate.

The experimental setup was prepared in triplicate as shown in Fig. 1. For each cell, two Teflon containers were built to enable water saturation of the test samples during the experiments. The concrete was poured into the Teflon molds, placing the C-steel container in the center. The core of the cell was composed of a steel container with a height of 45 mm and diameter of 20.64 mm. The internal Teflon container had a height of 100 mm and a diameter of 50 mm. The internal Teflon container surface area was about 140 cm^2^ and 5 holes with 0.7 mm diameter were randomly drilled through it to ensure saturation. The external Teflon container had a height of 160 mm and a diameter of 90 mm, and it was used as a container for soaking water, which was made by mixing 77 g of crushed concrete with 700 ml of MQ-water (Ultrapure Grade 1 water) for three days. Each experimental setup was weighed monthly to exclude leakage. All three cells were sealed to maintain anoxic conditions. A constant temperature of 80 ± 2 °C was imposed during the whole experiment. The corrosion potential was monitored using a Pt reference electrode. One of the experimental setups was opened for post-mortem characterization after 3, 7 and 12 months (hereafter referred as SC-3M, SC-7M, SC-12M with SC for Steel/Concrete). After disassembling the experimental setup, the C-steel containers were covered with adherent concrete, making direct characterization of steel surface difficult. The corrosion interfaces were thus investigated in cross-section by Scanning Electron Microscopy—Energy Dispersive X-ray Spectroscopy (SEM/EDX) and micro-Raman methods. The solutions were analyzed by Inductively Coupled Plasma Optical Emission Spectroscopy (ICP-OES) and Ion Chromatography (IC).

## Experimental results

### Corrosion potential

**Figure 2:**
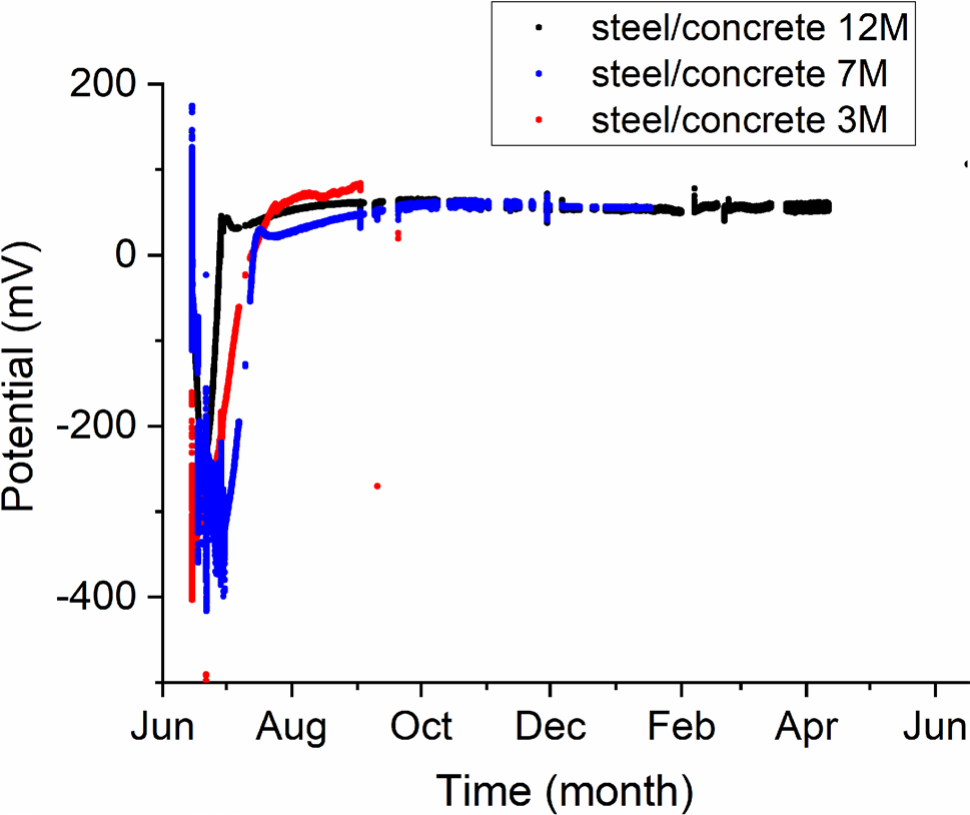
Evolution with time of the measured corrosion potentials on the standard hydrogen scale for the three cells at 80 °C.

Figure 2 gives the relationship of corrosion potential with months of exposure. The evolution of the corrosion potential measured on the three steel/concrete setups (3 M, 7 M, 12 M) follow the same characteristics at all stages of experiments. The strongly negative values during the first two months are linked to the corrosion of the C-steel. Afterwards, the potentials decrease to reach small constant positive values that are assumed to be driven by the formation of a passivation layer. Film passivation in less than 50 days is notable for all three setups.

### SEM/EDX analysis

**Figure 3:**
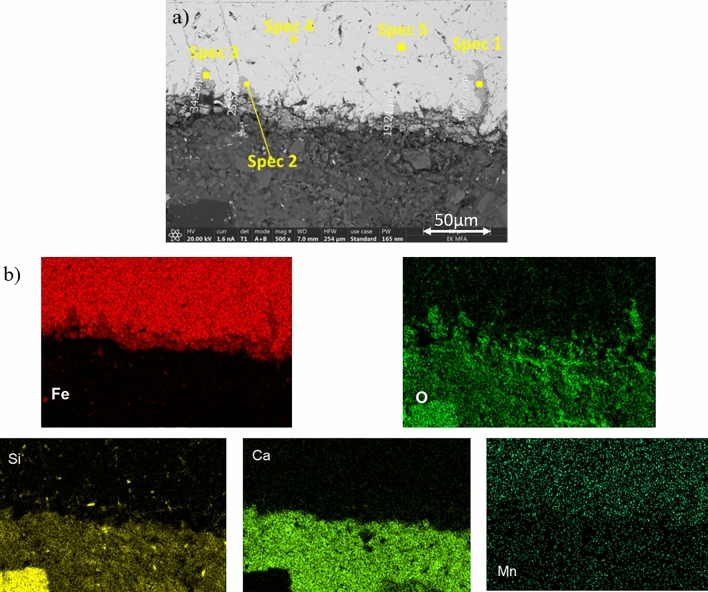
(**a**, **b**) SEM micrograph showing micro-cracks initiated in the steel in contact with concrete after 3 months (SC-3M sample) (**a**) and the corresponding elemental mapping of Fe, O, Si, Ca and Mn (**b**).

**Figure 4:**
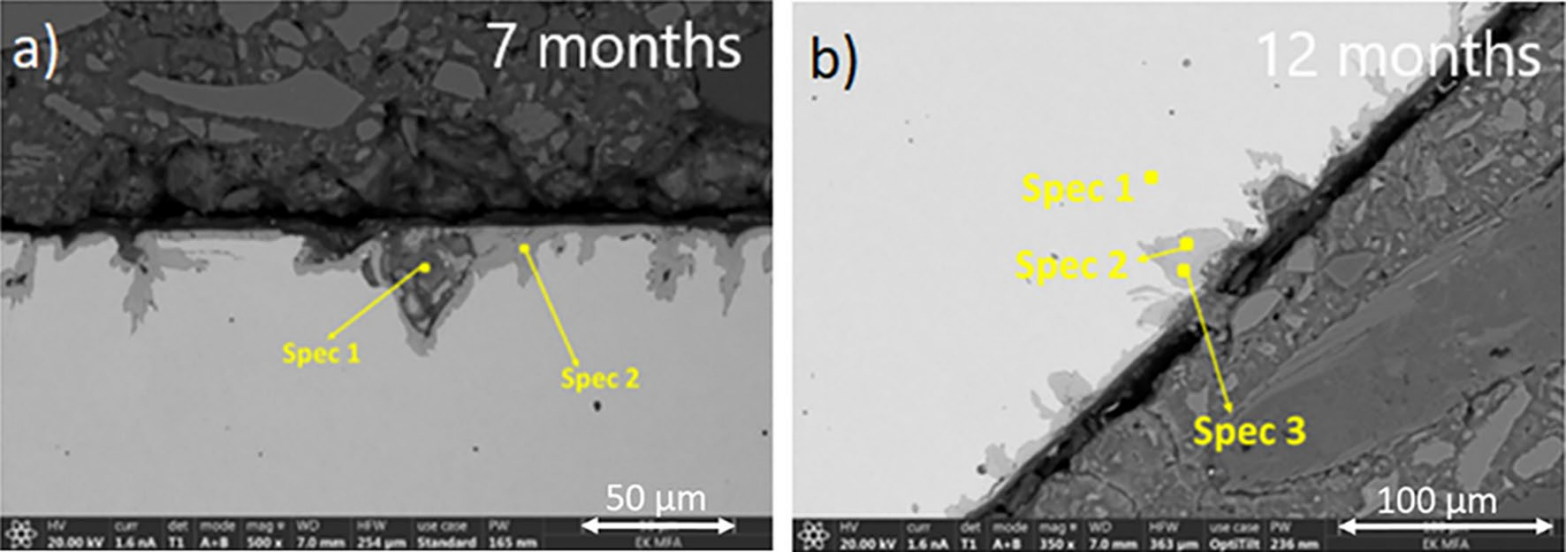
(**a**, **b**) SEM micrograph of the steel–concrete interface after 7 months (**a**) and 12 months (**b**).

Figure 3 presents SEM images of the steel/concrete interfaces after 3, 7, 12 months experimental time. There is no indication of voidage or free space in between the concrete and the steel. The pouring of the concrete had led to tight interfaces since the beginning of the experiment. On each of the three samples the formation of 20–60 µm long Fe-oxide ingrowths were detected. These SEM results show that micro-cracks appeared even in a short term (3 months) and could have initiated changes of the carbon steel surface (Fig. 3). However, these ingrowths remained relatively short and did not exceed 100 µm in length after 7 and 12 months (Fig. 4).

Table 2 gives the results of the SEM–EDX analyses for minerals at the corrosion interfaces after 3 months (SC-3M) based on the points marked in Fig. 3. Corrosion products consisting of Fe–O (spectra 1–3) can be distinguished from the C-steel (spectra 4 and 5), which is primarily composed of Fe. Table 3 gives the EDX analyses after 7 months with again a Fe–O signature typical of iron oxide corrosion products (spectra 2), but there is also a mixed signal composed of Ca–Al–Si–S and Fe oxide. Table 4 provides results similar to those of Table 2.

**Table 2:** SEM–EDX analyses of the corrosion products and the steel for the SC-3M sample (the locations of the spectra are given in Fig. 3a).

[at. %]	Spec 1 corr. prod.	Spec 2 corr. prod.	Spec 3 corr. prod.	Spec 4 C-steel	Spec 5 C-steel
O	47.09	44.01	47.64	–	–
Si	1.67	0.18	0.47	0.65	0.97
Ca	0.16	0.15	0.11	–	–
Mn	0.36	0.28	0.21	0.59	0.61
Fe	50.72	55.38	51.57	98.76	98.42
Total	100.00	100.00	100.00	100.00	100.00

**Table 3:** SEM–EDX analyses of the corrosion products and the steel for the SC-7M sample (see Fig. 4 a for location of analyses).

[at. %]	Spec 1 concrete/corr. prod.	Spec 2 corr. prod.
O	56.45	46.84
Mg	3.43	–
Al	2.41	–
Si	12.10	0.34
P	0.06	–
S	0.51	–
Cl	0.05	–
K	0.08	–
Ca	3.59	0.14
Ti	0.06	–
Mn	0.15	0.37
Fe	21.13	52.31
Total	100.00	100.00

**Table 4:** SEM–EDX analyses of the corrosion products and the steel for the SC-12M sample (see Fig. 4b).

[at. %]	Spec 1 C-steel	Spec 2 corr. prod.	Spec 3 corr. prod.
O	–	51.46	58.26
Si	0.46	–	–
Ca	–	0.08	0.12
Fe	99.54	48.46	41.62
Total	100.00	100.00	100.00

### Mineralogical phase identification

**Figure 5:**
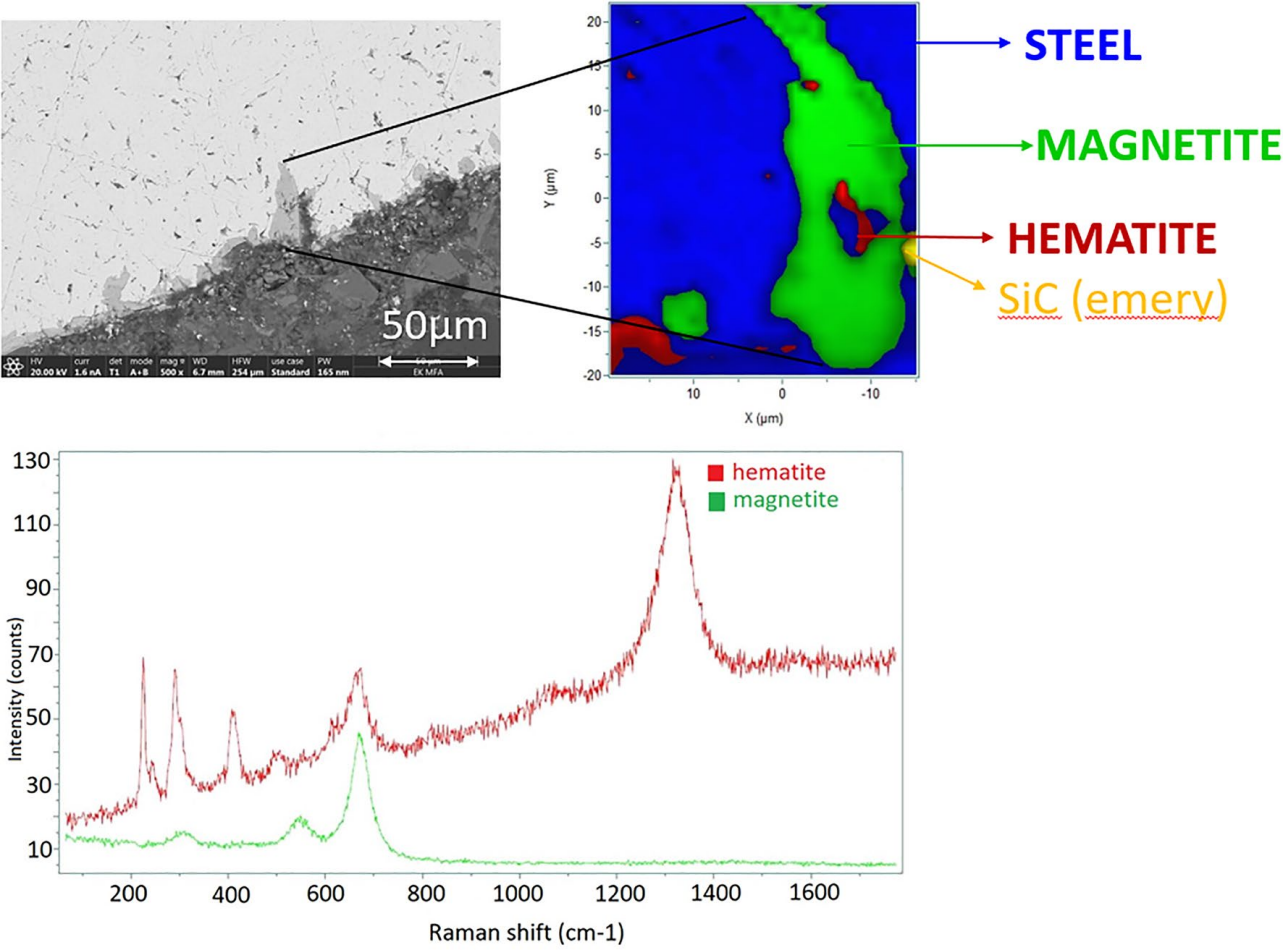
Micro-Raman mapping and characteristic spectra obtained after 3 months (SC-3M).

**Figure 6:**
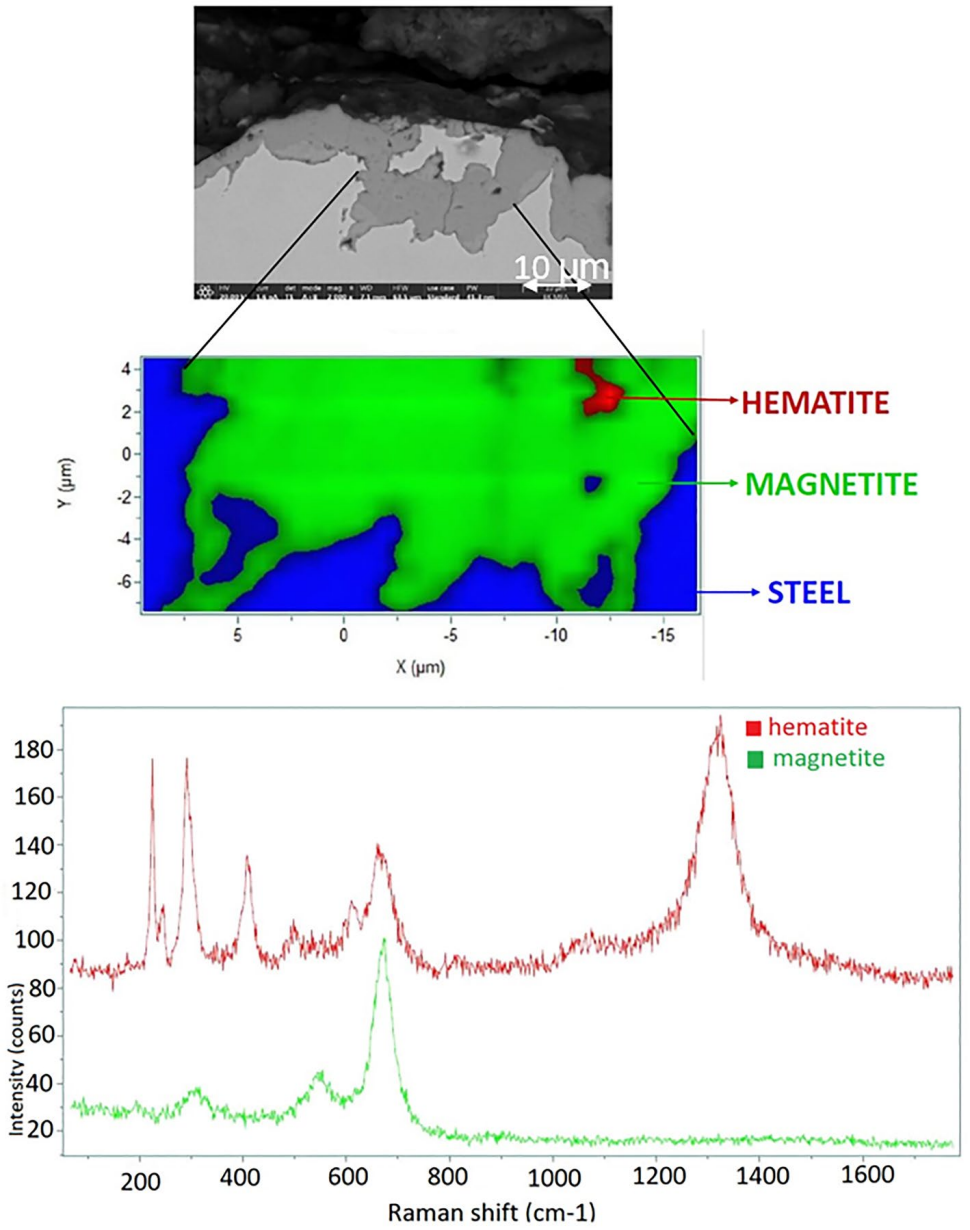
Micro-Raman mapping and characteristic spectra obtained after 7 months (SC-7M).

**Figure 7:**
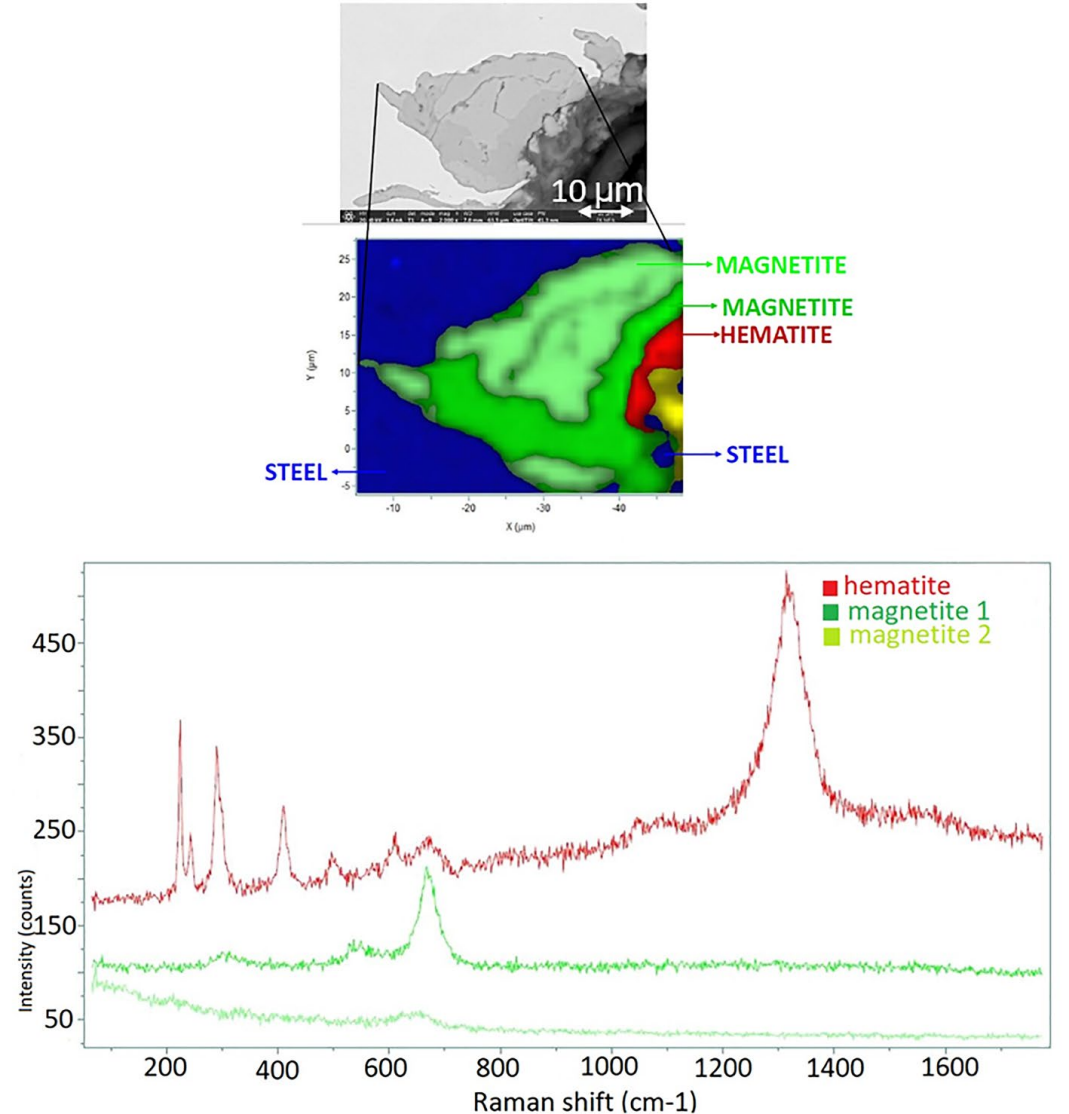
Micro-Raman mapping and characteristic spectra obtained after 12 months (SC-7M).

According to the results of the SEM–EDX, the corrosion process had an impact on the steel–concrete interfaces. With micro-Raman investigations mainly magnetite (Fe_3_O_4_) and hematite (α-Fe_2_O_3_) can be identified as corrosion products after 3 months (SC-3M sample Fig. 5) and 7 months (SC-7 M sample, Fig. 6). The corrosion protrusion is formed of magnetite, no other corrosion products could be identified by micro-Raman. After 12 months of experiments, no substantial change in the Raman spectra were observed (Fig. 7). Mainly magnetite (Fe_3_O_4_) and hematite (α-Fe_2_O_3_) were still detected. For all the exposure periods tested, magnetite was identified as the main corrosion product. No iron-carbonates or iron-sulfides were identified.

### Chemical analysis of aqueous solutions

The liquid phase (soaking water) was sampled from the external Teflon container after 3, 7 and 12 months. Ca, K, Mg, Na and Si concentrations were determined using inductively coupled plasma—optical emission spectrometry (ICP-OES), while ion chromatography (IC) was used for Cl^−^ and SO_4_^2−^ (Table 5).

**Table 5:** Evolution of the chemistry of the aqueous solution sampled in the external Teflon container (Fig. 1b).

Concentration [mg/l]	Ca	K	Mg	Na	Si	Fe	Al	Cl^-^	SO_4_^2−^
Soaking water	97	24	2	219	43	0.4	0.8	505	100
3 months	–	–	–	–	–	–	–	449	157
7 months	117	19	1	238	44	< DL^a^	< DL	505	115
12 months	135	26	18	299	47	< DL	< DL	618	145

^a^DL stands for detection limit.

The Na and Ca concentrations progressively, but moderately, increased with time. The concentration of K and Si did not change significantly. Higher Mg concentration was measured after 12 months. The evolution of chloride and sulfate concentrations were less smooth, but the increase after 12 months was near 25% for chloride and 50% for sulfate.

## Modeling results

### Initial hydrochemistry and mineralogy of the concrete

The reactive transport code HYTEC was used to model the initial geochemistry of the concrete as well as its evolution with temperature and time^20^. The objective was to obtain a reliable estimation of the chemical conditions within the system, specifically focusing on the interface between the steel and concrete. To the best of our knowledge this study represents the first attempt to model the CEM-II based concrete, which is relevant to Hungarian radioactive waste management. Additionally, phase stability diagrams (Eh–pH diagrams) were calculated to support the discussion.

The recipe of the PURAM concrete detailed in Table 1 was considered for the modelling. The oxide composition of the CEM II-B-S 42.5 N was taken from Lubloy et al.^21^ and Pizon et al.^22^. The proportions of the CEM II-B components were set at 73.5 wt.% CEM I clinker, 24.5 wt.% blast furnace slag and 2 wt.% anhydrite (CaSO_4_)^21^. The oxide compositions of the clinker and blast furnace slag^22^ are detailed in Table SI-1. The hydration of the CEM I clinker was assumed to be completed. The literature on blended cements with blast furnace slags shows that one half of the slag has reacted after one month of hydration and that the remaining half could take as long as several years at ambient temperature^23,24^. There was no information on the hydration and reactivity of the slag in the present work at 80 °C. As a first approximation, the present modeling considered that one half of the slag was hydrated. The limestone was considered as 100% CaCO_3_, the silica fume as 100% SiO_2_. The siliceous sand and gravel aggregates were assumed to be non-reactive during the time frame of the experiment and were not included in the modeling.

Table 6 gives an estimation of the initial reactive mineralogy of the concrete calculated at room temperature and 80 °C. The mineralogy is in line with the literature on clinker/slag cements^23–25^. The rather high amount of Al in the slag leads to calcium-(aluminate-)silicate-hydrates (C-(A)-S-H phases) instead of pure calcium-silicate-hydrates (C-S-H) ones. Al also promotes the formation of monocarboaluminate (AFm phases) and ettringite (AFt phase). Mg is mostly found in hydrotalcite. Portlandite and ettringite have been identified by thermogravimetric analysis in hydrated CEM II/B concretes^21^. In relation to the objective of this study, a sensitivity analysis was conducted to assess the impact of the ratio and degree of hydration of the slag on the mineralogy. In good agreement with the literature^23^, the results indicated that increasing slag hydration beyond the assumed 50% will increase the C-(A)-S-H content and progressively decrease the portlandite content up to zero. The increase of temperature from 20 to 80 °C mainly results in the full dissolution of ettringite (and partial dissolution of monocarboaluminate) to form monosulfoaluminate (and calcite) in agreement with study of Lothenbach et al.^26^.

**Table 6:** Calculated initial amounts of reactive hydrated phases of the concrete.

Solid phases^a^ [kg/dm^3^ of concrete]	Chemical formula^b^	T = 20 °C	T = 80 °C
Calcium (Aluminate) Silicate Hydrate	C1.6-A0.01-S-H	0.357	0.357
Portlandite^c^	Ca(OH)_2_	*0.044*	*0.044*
Ettringite	Ca_6_Al_2_(SO_4_)_3_(OH)_12_∙26H_2_O	0.031	0.000
Monocarboaluminate	Ca_4_Al_2_(CO_3_)(OH)_12_∙5H_2_O	0.054	0.028
Monosulfoaluminate	Ca_4_Al_2_(SO_4_)(OH)_12_∙6H_2_O	0.000	0.044
Hydrotalcite	Mg_4_Al_2_O_7_∙10H_2_O	0.025	0.025
Calcite	CaCO_3_	0.350	0.355

^a^The non-reactive sand and gravel aggregates were not assumed to be reactive phases; ^b^Thermoddem database; ^c^assuming that one half of the slag has been hydrated, further hydration would lead to the lack of portlandite.

Significant values are in [italics].

Table 7 provides for an estimation of the initial porewater chemistry of the concrete calculated at 80 °C in thermodynamic equilibrium with the reactive minerals of Table 6. The pH of young cement water, which represents the early composition of the cement porewater during the long-term operation of a repository, is about 11.9 at 80 °C. This high pH value is a maximum estimation driven by the dissolution of Na_2_O and K_2_O in water to form NaOH and KOH. The initial contents in Na_2_O and K_2_O were not accurately known. The modeling assumed that 50% of these alkali hydroxides were sorbed onto the C-(A-)S-H phases. The increase of temperature decreases the pH and significantly increases sulfate concentration in the porewater. In the current modeling approach, the sorption of sulfate onto C-(A-)S-H was not taken into account. However, it is important to note that if this sorption process were considered, the dissolved sulfate concentration would be reduced by approximately 50%. On the other hand, chloride, which can exacerbate steel corrosion, was assumed to be fully dissolved in the porewater without being influenced by sorption or a mineral phase. At both temperatures, the initial chloride concentration was approximately 1200 mg/l.

**Table 7:** Calculated pore water chemistry of the concrete at 20 °C and 80 °C.

Total concentration [mg/l]	T = 20 °C initial	T = 80 °C initial	T = 80 °C Steel interface, 12 months
pH	13.6	11.9	11.25
Na^+^	4350	4350	540
K^+^	7450 (750*)	7450 (750*)	605 (60)
Ca^2+^	35	29	149
Mg^2+^	0	0	0
Al^3+^	1	145	28
Cl^-^	1200	1200	563
HCO_3_^−^	14	27	1
SO_4_^2−^	256	3300	66
H_4_SiO_4_	5	9	1

Significant values are in [italics].

*K^+^ concentration required to fit the data of the Teflon container.

### Evolution of the aqueous chemistry with time

A second stage of reactive transport modeling was performed, based on the calculated initial chemistry and mineralogy of the concrete. The modeling grid of 2.5 mm resolution took into account the full experimental cell set-up in 2D-cylindrical geometry (Fig. SI-1 in supplementary information). The steel was not explicitly considered in the modeling, which focused on the concrete. Steel is a non-porous material that only reacts through its surface and steel corrosion was found to be very low in the present study. The porosity and effective diffusion coefficient of the concrete was set to 10% and 6 × 10^–12^ m^2^/s at 80 °C, respectively. These parameters were not measured directly but estimated as a reasonable set according to the literature and provided a good fit to the concentrations of the (almost) non-reactive elements Na^+^ and Cl^-^ measured in the external Teflon container. It is clear that the five drilled holes were sufficient to maintain water saturation, but they only induced a very low diffusive transfer rate. The effective diffusion coefficient of the Teflon container, 3 × 10^–13^ m^2^/s, could only be estimated/fitted on the same data set. A sensitivity analysis on the effective diffusion coefficient of the container is given in Fig. SI-2.

**Figure 8:**
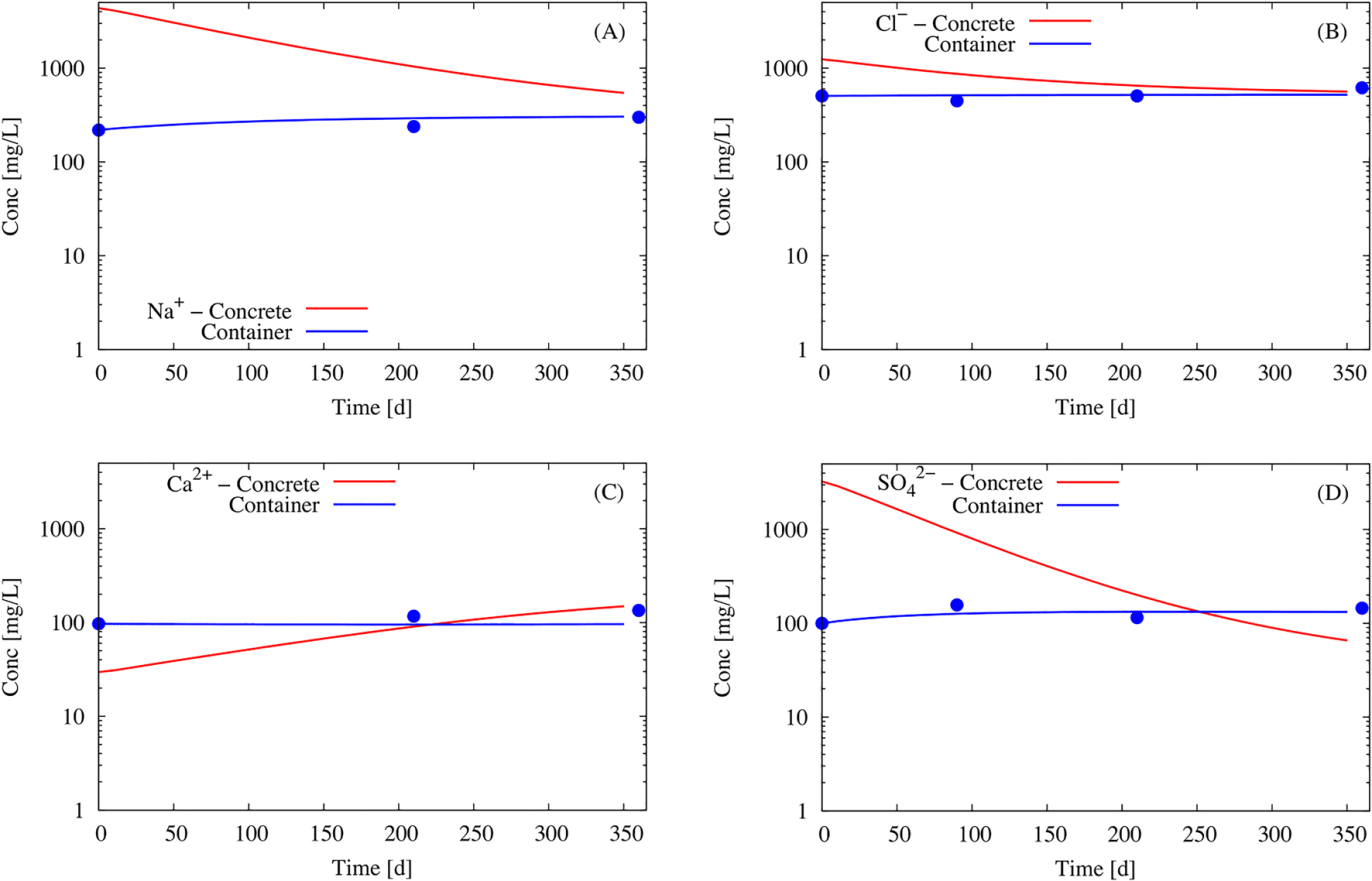
(**a**–**d**) Modeling of the time-dependent evolution of the chemistry of the concrete porewater (in red) and the chemistry of the solution in the external Teflon container shown in Fig. 1 (in blue). For comparison, experimental data from the container are included as blue dots.

**Figure 9:**
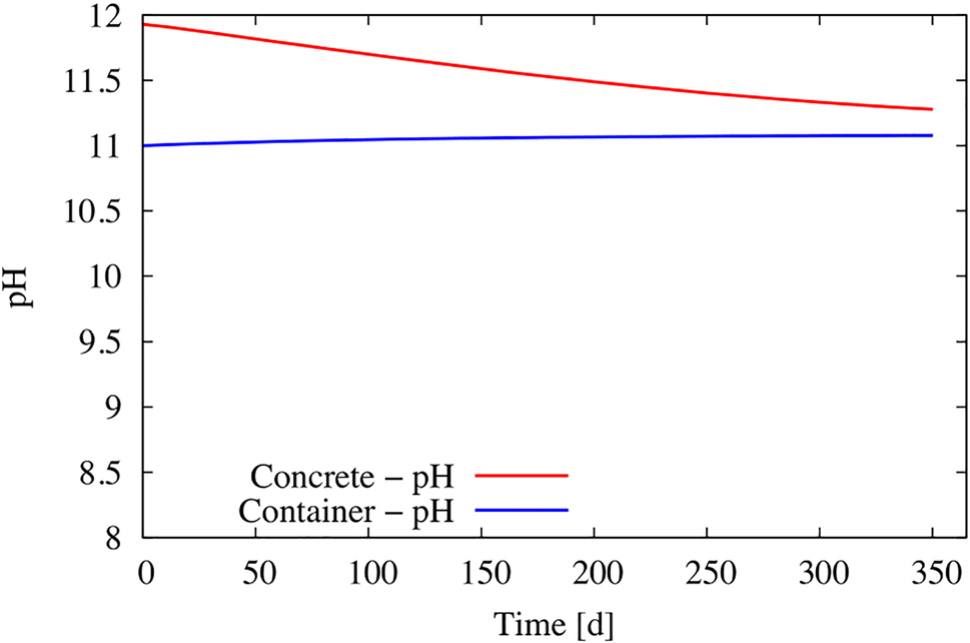
Modeling of the evolution with time of the pH of the porewater (in red) and the solution in the external Teflon container (in blue).

Figure 8a, b show the evolution over 1 year of the concentration of the weakly reactive elements Na^+^ and Cl^-^, which are only present as dissolved species in the model. Both concentrations are higher inside the concrete than in the soaking water. The gradient of concentration indicates diffusion from concrete towards the solution in the external container, which progressively led to uniform concentrations in the whole system. The good fit of the calculated concentrations after 1 year with the experimental data provides some confidence in the modeling. The decrease of the pH from 11.9 to about 11.2 inside the concrete (Fig. 9) is mainly due to the diffusion of the Na–K–OH plume towards the container. Despite diffusion, the pH remains quite high (pH about 11) in the concrete and hence also at the interface with steel. The changes over time in the concentrations of Ca^2+^ and SO_4_^2−^ (Fig. 8c, d, reactive species) are influenced by both diffusion and the equilibrium with cement minerals. This equilibrium is, in turn, dependent on the pH value. Table 7 gives the change in concentration of all the elements at the steel/concrete interface after 12 months. Such a relatively complex coupling between the chemical reactions and diffusion can only be handled with modeling. Again, the reasonably good agreement between the calculated and the experimental data lends credibility to the model.

The main effect on the mineralogy of the concrete is due to the temperature increase up to 80 °C. The globally weak diffusive transfer does not induce any significant change in the mineral contents. The only temperature-driven modification is the transformation of monocarboaluminate to monosulfoaluminate and calcite at the boundary of the concrete in contact with the solution in the external container (but not around the steel/concrete interface).

## Discussion on steel corrosion

### Corrosion products at 80 °C

Geochemical and reactive transport modeling of the concrete and experimental set-up provides a reasonably accurate estimation of the mineralogy and chemical conditions at the steel interface, considering factors such as temperature rise and diffusion within the system. These geochemical parameters, which are difficult to measure directly, were effectively taken into account. Although the mineralogy remains relatively stable throughout the process, the chemistry of the porewater undergoes some changes, yet remains predominantly highly alkaline overall. The redox conditions induced by the concrete itself are currently uncertain. According to the literature, reducing conditions are predicted by thermodynamic modelling of slag cements^15^. Indeed, blast furnace slags are rapidly cooled by-products from the reduction of iron ore in the blast furnace and they contain reducing Fe(0) or Fe(II) in the solid phase^27^. Furthermore, considering the anoxic nature of the setup, it is expected that reducing conditions would be prevalent.

**Figure 10:**
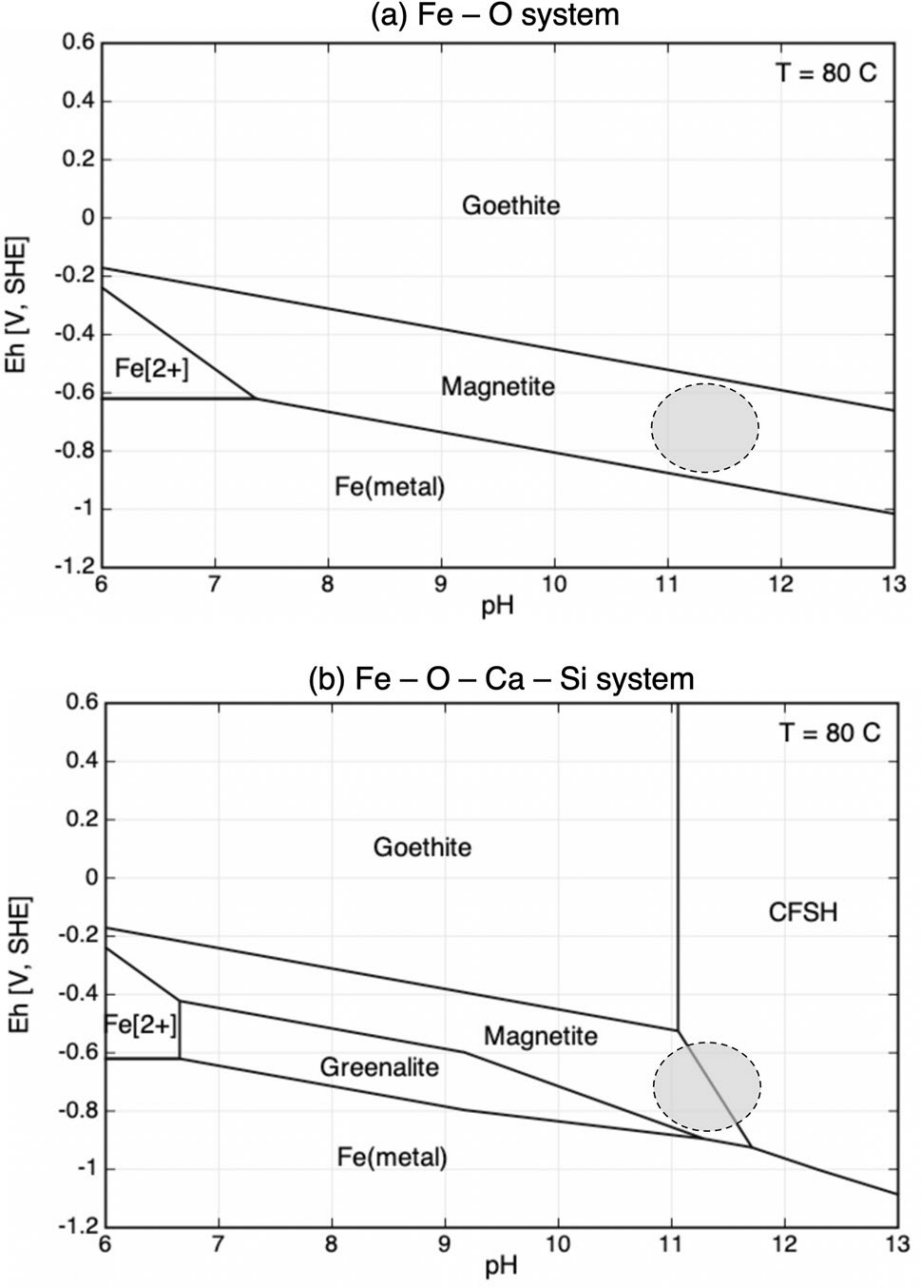
(**a**, **b**) Phase stability diagrams of possible corrosion products of C-steel in slightly to strongly alkaline chemical environments (CHESS/HYTEC code, Thermoddem database, section “Modeling code and database”); activity Fe^2+^  = 5 × 10^–5^, activity Ca^2+^  = 5 × 10^–4^, total H_4_SiO_4_ = 1 × 10^–5^, temperature = 80 °C; the grey zones stand for likely pH–Eh conditions in the present steel/concrete system.

Figure 10 shows some possible corrosion products that may form at the steel/concrete interface for the calculated chemical conditions. The grey zone on the pH-Eh diagram of the Fe–O system corresponds to competition between goethite (Fe(III) oxyhydroxide, FeOOH) and magnetite (Fe(III)-Fe(II) oxide, Fe_3_O_4_) (Fig. 10a). It should be noted that hematite (Fe(III) oxide, Fe_2_O_3_) will replace goethite in the same diagram if included in the system. If the system is further complicated by introducing Ca and Si (Fig. 10b), Fe siliceous hydrogarnet (Fe(III) C-F-S-H, Ca_3_Fe_2_(SiO_4_)_0.84_(OH)_8.64_) can become the main corrosion product instead of magnetite at high alkaline pH and mildly reducing to oxidizing redox potential. Fe(II)-silicates corrosion products such as greenalite (Fe_3_Si_2_O_5_(OH)_4_) can only precipitate for pH lower than 10.5 at 80 °C and reducing conditions.

Magnetite was clearly identified as the main corrosion product by micro-Raman. The SEM–EDX analyses in Fe and O of corrosion products are also consistent with magnetite. Magnetite is usually the main product formed during the anoxic corrosion of carbon steel^5,14,16,28^. No other corrosion products, such as Fe-silicate or Fe-sulfide, were identified by this technique. Magnetite remained stable over the 12 month test period and did not convert to iron silicates and/or iron sulfides as it has been observed in cementitious/bentonite grout at lower pH^29^. Hematite was identified in most cross-sections, usually close to the steel surface and always in relatively low quantity compared to magnetite. It is possible that hematite was present since the beginning of the experiment as mill scale, or else due to the transformation of early oxic corrosion products during the preparation of the experimental setups and then heating to 80 °C.

Fe-siliceous hydrogarnet CFSH is one of the most stable iron phases in hydrated cement^15,23^. The SEM–EDX spectrum 1 of Table 4 indicates a mixed signature between concrete and iron oxide elemental composition. The analysis may correspond to a Fe-siliceous hydrogarnet, but it is most probably an intrusion of concrete in a crack within the steel. It seems that in the present system magnetite formed more quickly on the steel surface than CFSH formation and was controlled by Ca and Si diffusion from the concrete. Chomat et al.^16^ have also seen Fe-enriched layers at the concrete interface with steel. Pally et al.^18^ observed hydroandratite (CFSH like phase) deposits on the iron plate in cementitious solutions at 80 °C, but in their experimental set-up diffusion could have been facilitated by direct contact of the steel with the synthetic solution and not the cement paste.

### Corrosion mechanism and rate

Microstructural characterization showed strong similarities in the corrosion patterns in 3, 7 and 12-months samples. Magnetite was found all along the steel surface although the formation of 20–60 µm long Fe-oxide ingrowths were also identified. The literature states that the carbon steel surface is likely to be passivated due to the formation of a stable magnetite film^5,16^. This is in line with the short transient stage of negative corrosion potential measured in the three cells for 50 days at maximum. The occurrence of a uniform passive corrosion mechanism is, therefore, likely.

A mean corrosion rate was derived from observed thicknesses during SEM analysis and from the obtained corrosion potential curve (estimating that the altered layer was formed only in the first 50 days). In this study the estimated corrosion rate was of the order of magnitude of 10 μm/year. Similar values of early corrosion rates have been found for CEM I and the corrosion rates usually decrease exponentially with time to reach rates smaller than 0.01 µm/year within a year^17^. The long-term corrosion rate could not be determined in the present experiments but was clearly much lower than 10 μm/year.

There was no trace of any localized corrosion. The moderated aqueous concentrations of chloride (500–1000 mg/l) had no effect.

## Conclusions

The current experimental setup examined the combined chemical evolution of two major components, namely the waste canister and the concrete buffer, used in deep geologic disposal at a temperature of 80 °C. The materials chosen for analysis, S235JR carbon steel and CEM II/B based concrete, are commonly used as reference materials in the Hungarian disposal concepts. However, the findings are applicable to other disposal systems and engineering barriers as well. During the experiments, it was observed that a uniform corrosion process occurred, resulting in the rapid passivation of the C-steel cylinders. Throughout the entire 12-month duration of the experiment, magnetite was identified as the primary corrosion product, even after only 3 months of exposure. Modeling revealed that the increase in temperature led to an increase in sulfate concentration due to the dissolution of ettringite and that diffusion of the concrete porewater into the external container led to a slight decrease in pH and chloride concentrations at the cement/steel interface. However, these chemical changes did not significantly affect the passivation corrosion process. Although the formation of Fe-siliceous hydrogarnets was thermodynamically possible at 80 °C, it did not hinder the formation of magnetite. The results show that the passivation of the containers under the tested conditions is a promising result for further investigations.

## Methods

### Corrosion potential measurements

Corrosion potential measurements were made by recording the potential difference between the studied material and a calomel reference electrode. If the corrosion potential of a metal increases with time (i.e. changes in a positive direction), then protection of the surface occurs if a passive film develops on the surface, and corrosion slows down. The oxidation–reduction potential of the test environment was monitored using a platinum wire that was embedded within the concrete and C-steel wire (also S235JR) that was spot welded on the container. Measurements were taken at 20-s intervals. Initially, the potential between the platinum wire and a calomel electrode was checked using a Metrohm Autolab PGStat204 potentiostat. The measurements were conducted in the soaking water under specific conditions, namely at pH 11.9 and a temperature of 22.8 °C. To ensure accurate interpretation and comparison of the results, the obtained potential was then adjusted using an offset correction with a value based on the potential values of common reference electrode conversions. This correction was applied to align the results to the standard hydrogen electrode (SHE) scale. By making this adjustment, the Eh potential values can be properly referenced and analyzed in relation to other electrochemical systems or experiments conducted using the SHE scale.

### Scanning electron microscopy measurements

The composition and nature of alteration products formed on the steel and within the concrete were investigated by scanning electron microscopy/energy dispersive X-ray microanalysis (SEM/EDX). The measurements were performed at 20 kV and 1.6 nA, using a Thermo Scientific Scios2 dual beam microscope, Oxford X-maxn 20 SDD EDX^30^.

### Micro-Raman spectroscopy investigations

Micro-Raman investigations were performed to identify the corrosion products. The analyses were carried out using a HORIBA JobinYvon LabRAM HR 800 Raman microspectrometer. A frequency doubled Nd-YAG green laser with a 532 nm excitation wavelength was used to illuminate the samples, by applying ~ 0.2 mW to the sample surface. An OLYMPUS 50 × (Numerical Aperture = 0.6) or a 100 × (Numerical Aperture = 0.9) objective was used to focus the laser. For Raman mapping, a 100 μm confocal pinhole, 600 grooves/mm optical grating, 4–10 s cumulated exposition time were used. The spectral resolution of measurements was 3.0/cm. The step size of the Raman maps varied between 1 and 0.5 μm for magnifications 50× and 100× , respectively^30^.

### Inductively coupled plasma optical emission spectroscopy and ion chromatography

ICP-OES measurements were carried out on a Perkin Elmer Avio 200 instrument. The leachates were filtered through a cellulose acetate membrane (pore size > 220 nm) and acidified with 20 μl of ultrapure HNO_3_. All elements were tested in radial view using 1 ppm of Yttrium as internal standard and a calibration of 4 points was applied for all measured elements (0.01, 0.1, 1 and 10 ppm). The power of the generator was set to 1200 W during the measurements and the flow rate of the plasma argon was 12 l/min. IC analyses were performed on Thermo Scientific Dionex Aquion equipment with 4.5 mM Na_2_CO_3_/0.8 mM NaHCO_3_ eluent composition and at a flowrate of 0.25 ml/min. The separation of the components was carried out using a Dionex IonPac AS23 2 mm × 250 mm analytical column coupled with a Dionex AERS 500 Carbonate electrochemical suppressor.

### Modeling code and database

The reactive transport code HYTEC^20^ was used and phase stability diagrams (Eh-pH diagrams) were calculated with CHESS, the geochemical module of HYTEC. All calculations were performed at thermodynamic equilibrium. To account for activity corrections implementation of the truncated-Davies model was considered. The Thermoddem database [^31^, version 12-2019] was selected to provide accurate thermodynamic constants at both 20 and 80 °C. This comprehensive database encompasses a wide range of minerals that are pertinent to cement phases and corrosion products. In addition, the formation constants of the Fe(III)-siliceous hydrogarnet C-F-S-H (Ca_3_Fe_2_(SiO_4_)_0.84_(OH)_8.64_) were incorporated into the database^32^.

### Supplementary Information

Supplementary Information.

## Data Availability

The data related to the present study can be obtained from the corresponding author M. Fábián (fabian.margit@ek-cer.hu) upon personal request.
