# Supplementary material for: Interactions between C-steel and blended cement in concrete under radwaste repository conditions at 80 °C
Source: Sci Rep. 2023 Sep 16;13:15372. doi: 10.1038/s41598-023-42645-6 (PMC10505235; doi:10.1038/s41598-023-42645-6)
Supplement: Supplementary file 1 — Supplementary Information. [file 41598_2023_42645_MOESM1_ESM.pdf]

**Interactions between C-steel and blended cement in concrete under radwaste repository conditions at 80 °C**

Margit Fabian[1]*, Otto Czompoly[1], Istvan Tolnai[1], Laurent De Windt[2]

[1]*Centre for Energy Research, Konkoly Thege st 29-33, 1121 Budapest, Hungary, fabian.margit@ek-cer.hu*

[2]*Mines Paris, PSL University, Centre for geosciences and geoengineering, 77300 Fontainebleau, France*

## Supplementary information

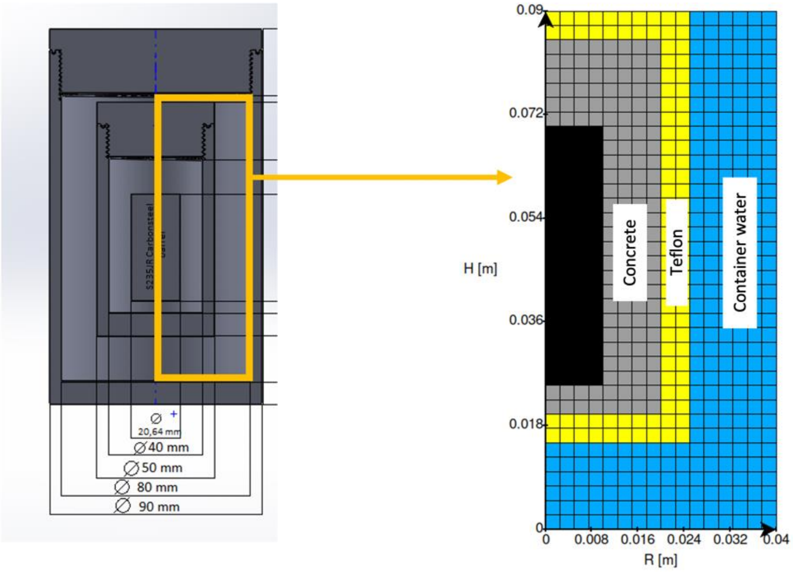


**Fig. SI-1** Modeling grid of 36 (H) × 16 (R) cells simulating the experimental cell in cylindrical symmetry.

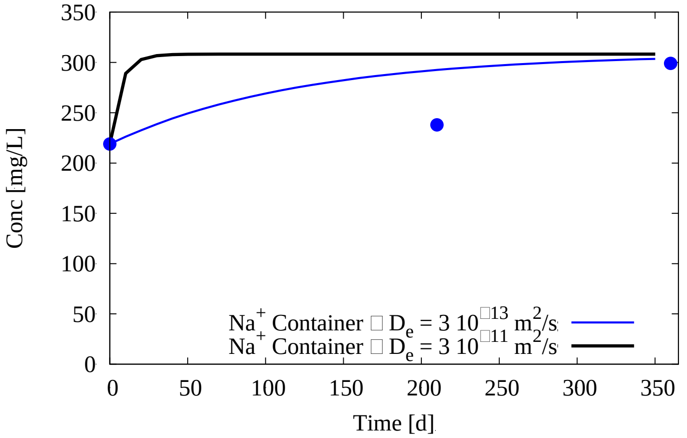


**Fig. SI-2** Effect of the diffusion coefficient on the time evolution of the assumed non-reactive $Na^+$ total concentration; the experimental data of the solution in the external container are added (blue circles) for the sake of comparison.

**Table SI-1.** Oxide composition of the clinker and blast furnace slag considered in the modeling [21, 22].

| **Composition [wt. %]** | **Clinker** | **Slag** |
|---|---|---|
| $SiO_2$ | 21.3 | 37.4 |
| $Al_2O_3$ | 5.0 | 7.3 |
| $Fe_2O_3$ | 3.4 | 1.2 |
| CaO | 64.8 | 43.9 |
| MgO | 2.1 | 5.7 |
| $K_2O$ | 1.0 | 0.56 |
| $Na_2O$ | 0.11 | 0.55 |
| Cl | 0.03 | 0.03 |
| $CaSO_4$ | 2.50 | - |
